# A Bayesian Mixture Model approach to expected possession values in rugby league

**DOI:** 10.1371/journal.pone.0308222

**Published:** 2024-11-21

**Authors:** Thomas Sawczuk, Anna Palczewska, Ben Jones, Jan Palczewski

**Affiliations:** 1 School of Built Environment, Engineering and Computing, Leeds Beckett University, Leeds, United Kingdom; 2 Carnegie Applied Rugby Research (CARR) Centre, Carnegie School of Sport, Leeds Beckett University, Leeds, United Kingdom; 3 England Performance Unit, The Rugby Football League, Leeds, United Kingdom; 4 Premiership Rugby, London, United Kingdom; 5 Division of Physiological Sciences and Health through Physical Activity, Lifestyle and Sport Research Centre, Faculty of Health Sciences, University of Cape Town, Cape Town, South Africa; 6 School of Behavioural and Health Sciences, Faculty of Health Sciences, Australian Catholic University, Brisbane, Australia; 7 School of Mathematics, University of Leeds, Leeds, United Kingdom; Portugal Football School, Portuguese Football Federation, PORTUGAL

## Abstract

This study aimed to introduce a novel Bayesian Mixture Model approach to the development of an EPV model in rugby league, which could produce a smooth pitch surface and estimate individual possession outcome probabilities. 99,966 observations from the 2021 Super League season were used. A set of 33 centres (30 in the field of play, 3 in the opposition try area) were located across the pitch. Each centre held the probability of five possession outcomes occurring (converted/unconverted try, penalty, drop goal and no points). Probabilities at each centre were interpolated to all locations on the pitch and estimated using a Bayesian approach. An EPV measure was derived from the possession outcome probabilities and their points value. The model produced a smooth pitch surface, which was able to provide different possession outcome probabilities and EPVs for every location on the pitch. Differences between team attacking and defensive plots were visualised and an actual vs expected player rating system was developed. The model provides significantly more flexibility than previous zonal approaches, allowing much more insightful results to be obtained. It could easily be adapted to other sports with similar data structures.

## Introduction

The use of advanced statistical and machine learning methods to evaluate player and team performances through expected possession value (EPV) models is growing in sport [1–6]. EPV models value every action and/or location on the field of play with respect to its point-scoring potential. These models represent an extension of the expected goals metric, which has become prominent in football [7] and ice hockey [8]. However, although insightful models have been produced in sports with high quality (i.e. match event and player tracking data) and quantity (i.e. millions of observations) data availability [1–4], the models in low data availability sports (e.g. rugby league, netball or hockey) are much more limited [5, 6]. In these sports, the adoption of different advanced statistical approaches could improve the quality and predictive power of models and the usefulness of the results obtained.

To date, two EPV models have been published within rugby league [5, 6]. Both studies utilised a Markov Reward Process (MRP) approach [9], which required data to be aggregated and valued equally within a set of zones. Aggregating data into zones is a common practice within spatial analyses across multiple domains [10, 11] and has been used successfully in basketball [1]. Unfortunately, rugby league has a much larger playing surface than basketball, which limits the usefulness of this method when attempting to evaluate team and player performances. For example, Ref. [12] found that in a worst-case scenario, a player could run 60m forward on the pitch from their team’s 10m line to the opposition 30m line and receive no positive value for their action. A second limitation of the MRP approach is that it aggregates all scoring outcomes into a single value. In rugby league where there are five scoring outcomes, it is possible that this could result in the loss of valuable tactical information. For example, a team may be more likely to score converted tries on one side of the pitch, but unconverted tries on the other. Similarly, they may be more likely to score drop goals than penalty goals from a specific area on the pitch. If the probability of each scoring outcome could be modelled individually, before being combined to produce an EPV measure, it may be possible to glean specific tactical insights from the data. There is therefore scope for the adoption of a novel approach to EPV modelling, which allows a smooth pitch surface to be calculated for each possession outcome probability in a low data availability sport.

One method through which a smooth pitch surface could be estimated for individual possession outcomes is through a Bayesian Mixture Model [13]. Recently, Bayesian approaches have been used effectively to analyse spatial data in sport [1, 14]. Bayesian analysis uses an evidence-based approach within the estimation of model parameters. This allows it to calculate certainty and uncertainty in parameter estimates conditional on the volume of evidence supporting the model’s conclusions. Furthermore, the use of prior distributions allows the model to understand likely parameter values before the model fitting process begins. In a low data availability sport, this prior understanding is extremely advantageous compared to machine learning and frequentist statistical approaches, which randomly initialise parameter values and thus require more data to provide accurate parameter estimates. A Mixture Model is a probabilistic model, which is comprised of a set of mixture components and weights. The weights describe the relationship between the data and the mixture components. They provide an alternative method of aggregating a rugby league pitch’s spatial data around a set of mixture components (which could be considered as centres on the pitch), allowing values to be produced for each individual *x*, *y* location, rather than aggregating their values within a set of zones. Furthermore, these mixture components can estimate multiple categorical values concurrently providing a methodology through which individual possession outcome probabilities could be estimated in rugby league.

The primary aim of this study was to introduce a novel Bayesian Mixture Model approach to EPV modelling. This methodology improved upon previous MRP approaches to EPV models in low data availability sports by producing a smooth pitch surface and estimating individual possession outcome probabilities. A secondary aim of the study was to show how the model could be used to identify differences in teams’ attacking and defending performances and evaluate player performances.

## Methodology

Event level match-play data were obtained from Opta (Stats Perform, London, UK) for all 138 matches of the 2021 Super League season. In total, 557,050 match events were recorded, covering a range of actions (e.g. passes, kicks and runs) and descriptive data (e.g. video referee reviews, yellow and red cards). Across the season, 1001 tries were scored (768 successful conversion kicks, 233 unsuccessful conversion kicks), 175 penalty goals were attempted (158 successful, 17 unsuccessful) and 83 drop goals were attempted (37 successful, 46 unsuccessful). Prior to analysis, informed consent was obtained and ethics approval was provided by a sub-ethics committee at Leeds Beckett University. No direct informed consent was required as anonymised secondary data was used, but consent to use the anonymised data was provided by the gatekeeper to the data at Rugby Football League and via the license agreement with the data handler (Opta).

### Data preprocessing

For the purposes of this study, only the location of actions performed by the attacking team were required. The type of action (e.g. catch, pass, run) was not considered so if two or more consecutive actions were present in the same location, they were merged into a single entry for that location. The removal of defensive actions, incomplete actions, descriptive data and multiple action/location codings resulted in a final dataset of 99,966 actions. Appendix A in S1 Appendix provides further details surrounding this process.

Sequences of action locations completed by the same team were grouped together as possessions. A possession began when a team successfully gained possession of the ball and ended due to a handover, loss of possession caused by an error/foul play, points being scored or a goal kick attempt. It was therefore possible for an attacking possession to encompass more plays than the typical attacking set of 6 tackles if an error/foul was made by the opposition team.

Five possession outcomes were defined and treated as discrete categories: converted try; unconverted try; penalty goal; drop goal; and no try. These possession outcomes are the same as those used in previous studies [5, 6] and identify the result of the possession upon its completion. By treating the possession outcomes categorically it was possible to estimate individual possession outcome probabilities, improving upon the previous approach of treating each outcome numerically and aggregating the results into a single value [5, 6].

The data were organised into 25 subsets to allow the estimation of possession outcome probabilities at two levels: whole league; and team attacking/defensive. The whole league data subset was represented by all 99,966 observations. 12 team attacking (median 8105 actions per team, interquartile range 7596–8937) and 12 team defensive (median 8077 actions per team, interquartile range 7878–8700) subsets were also produced. These subsets comprised only actions performed by the relevant team (attacking) or only actions performed by all teams against the relevant team (defending). Table 1 provides a sample possession from the dataset.

**Table 1:** Sample possession used in this study. Data includes the teams involved in the possession, the player ID, the *x*, *y* coordinates of the action, the possession number (PosNum) and the possession outcome (PosCat; in this case no try for all rows).

Attacking Team	Defending Team	Player ID	*x*	*y*	PosNum	PosCat
Team A	Team B	3107	9	4	1	0
Team A	Team B	21716	9	6	1	0
Team A	Team B	1983	14	11	1	0
Team A	Team B	2904	22	13	1	0
Team A	Team B	11439	12	12	1	0
Team A	Team B	21795	37	16	1	0
Team A	Team B	2904	54	35	1	0

### A Bayesian Mixture Model approach

For the EPV model, a Bayesian Mixture Model approach was used to provide a smooth pitch surface and estimate individual possession outcome probabilities in rugby league. The model provides the probability of each possession outcome (*s* ∈ {converted try, unconverted try, penalty goal, drop goal, no try}) for any location on the pitch via a mixture of components corresponding to ‘centres’ on the pitch. The probability *P*(*s*; *x*, *y*) of possession outcome *s* at location *x*, *y* is calculated as a weighted average of probabilities *P*_*k*_(*s*) at all centres on the pitch via the formula

$$\begin{array}{c} P\left(s;x,y\right)=\sum_{k}z_{k}\left(x,y\right)P_{k}\left(s\right), \end{array}$$ (1)

where *z*_*k*_(*x*, *y*) is the weight corresponding to the location (*x*, *y*) and *k*-th centre and *P*_*k*_(*s*) is the probability of possession outcome *s* at the centre *k*. The location of centres and the calculation of weights is described in Section *Centre Weights*.

The likelihood function for a set of data, $D={\left(x_{i},y_{i},s_{i}\right)}_{i=1}^{n}$, is given by:

$$P\left(D|\left(P_{k}\right)\right)=\prod_{i=1}^{n}P\left(s_{i};x_{i},y_{i}\right)=\prod_{i=1}^{n}\sum_{k}z_{k}\left(x_{i},y_{i}\right)P_{k}\left(s_{i}\right),$$

where (*P*_*k*_) is the sequence of probability vectors for centres on the pitch (i.e. the probability of each possession outcome occurring at each centre). In Bayesian modelling, the prior distribution of (*P*_*k*_) is defined by the user and the posterior distribution (*P*_*k*_) given the data *D*, i.e., *P*((*P*_*k*_)|*D*) is studied after it has been produced by the model.

The prior distribution for (*P*_*k*_) was defined to be independent between centres. At each centre *k*, the vector of possession outcome probabilities (*P*_*k*_(*s*), *s* ∈ {converted try, unconverted try, penalty goal, drop goal, no try}) followed the Dirichlet distribution with parameter *α*_*k*_ (which is a vector of positive reals):

$$P_{k}∼\text{Dirichlet}\left(\alpha_{k}\right).$$

The Dirichlet distribution’s output is a vector of probabilities. As it is only possible for one possession outcome to occur every possession in rugby league, this distribution was ideally suited as the prior for possession outcome probabilities.

The posterior distribution *P*((*P*_*k*_)|*D*) is intractable. Therefore, Markov Chain Monte Carlo (MCMC) sampling was used to generate samples from the posterior distribution of probabilities *P*_*k*_ at each centre. Those samples were used to draw conclusions on the posterior distribution of probabilities on the pitch. Their mean (the mathematical expectation of the posterior distribution) will be denoted by $P_{k}^{\mu }\left(s\right)$ and *P*^*μ*^(*s*; *x*, *y*), in keeping with the above notation.

### Centre weights

**Fig 1:**
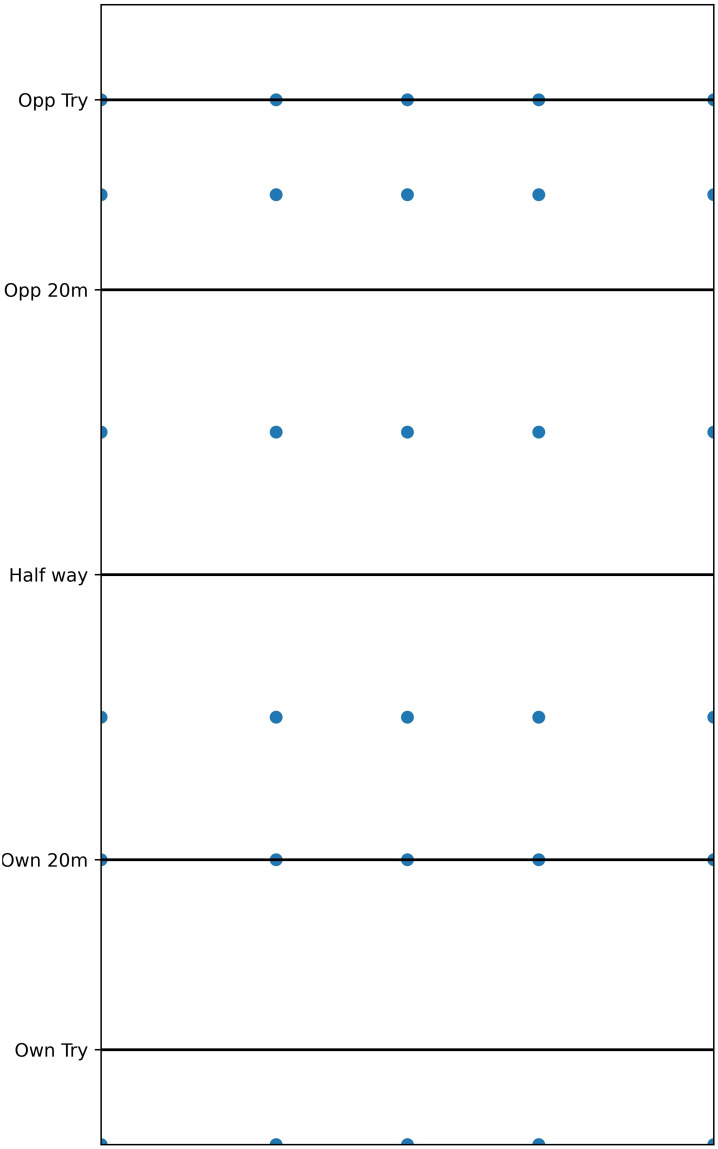
Location of the 30 field of play centres used in this study. Three opposition try area centres (not plotted) were included at *x* ∈ {0, 35, 70}, equivalent to the left, middle and right centres in the field of play. No *y* coordinate was considered for opposition try area centres.

After consultation with professional experts, 33 centres were placed around the pitch. 30 centres were located in the field of play, uniformly positioned at *x* ∈ {0, 20, 35, 50, 70} and *y* ∈ {-10, 20, 35, 65, 90, 100}. These locations were chosen to ensure that every location within the field of play would fall within a “zone” defined by four centres. Fig 1 plots the centre locations for the field of play. 3 centres were located in the opposition try area (*x* ∈ {0, 35, 70}). No *y* coordinate was considered for centres in the opposition try area as the actions players choose in this area are not usually influenced by their *y* coordinate. The field of play and opposition try area centres were evaluated separately. This decision was made due to the different player behaviours that are observed in the two areas: in the field of play, players are equally likely to choose different actions dependent on the game situation; in the opposition try area, players will attempt to ground the ball for a try as soon as possible irrespective of the game situation.

Each *x*, *y* location on the pitch was assigned 33 weights, which described their relationship with the 33 centres in the model. 30 weights were calculated in the field of play using bi-linear interpolation, 3 weights were calculated in the opposition try area using linear interpolation. In line with the assumption of independence between the two areas of the pitch, any location in the field of play was automatically given weights of 0 for the opposition try area centres; any location in the opposition try area was automatically given weights of 0 for the field of play centres.

Locations in the field of play had four non-zero weights. The value of the weights for each *x*, *y* location in the field of play was derived from the distance between the *x*, *y* location and the four centres surrounding it in a quadrilateral shape. Denoting the coordinates of these centres by (*x*_1_, *y*_1_), (*x*_1_, *y*_2_), (*x*_2_, *y*_1_) and (*x*_2_, *y*_2_), the weights *z*_11_, *z*_12_, *z*_21_ and *z*_22_ for these centres for location *x*, *y* were calculated as follows

$$\begin{array}{c} \begin{array}{cc} z_{11}=\frac{\left(x_{2}-x\right)\left(y_{2}-y\right)}{\left(x_{2}-x_{1}\right)\left(y_{2}-y_{1}\right)}, & \;z_{12}=\frac{\left(x_{2}-x\right)\left(y-y_{1}\right)}{\left(x_{2}-x_{1}\right)\left(y_{2}-y_{1}\right)},\\ z_{21}=\frac{\left(x-x_{1}\right)\left(y_{2}-y\right)}{\left(x_{2}-x_{1}\right)\left(y_{2}-y_{1}\right)}, & \;z_{22}=\frac{\left(x-x_{1}\right)\left(y-y_{1}\right)}{\left(x_{2}-x_{1}\right)\left(y_{2}-y_{1}\right)}. \end{array} \end{array}$$ (2)

The remaining centres were assigned a weight of 0.

Locations in the opposition try area had a maximum of two non-zero weights. Here, only the *x* location of the centres (*x* ∈ {0, 35, 70}) was considered so the weights were derived from the distance between the *x* coordinate of the action location and the *x* coordinate of the centre. For an *x*, *y* location in the opposition try area, linear interpolation between the two closest centres *x*_1_, *x*_2_, with *x*_2_ > *x*_1_, from the above set of three, was used to calculate two weights *z*_1_, *z*_2_; the weight of the remaining centre was set to 0. The non-zero weights were given by:

$$\begin{array}{c} \begin{array}{cc} z_{1}=\frac{x_{2}-x}{x_{2}-x_{1}}, & \;z_{2}=\frac{x-x_{0}}{x_{2}-x_{1}}. \end{array} \end{array}$$ (3)

### EPV calculation

The EPV for a location *x*, *y* was derived from the posterior distribution of possession outcome probabilities. It was calculated using the mean probability of each possession outcome at each centre and the true points scoring values:

$$\begin{array}{c} {\text{EPV}}_{\left(x,y\right)}=\sum_{s\in S}P^{\mu }\left(s;x,y\right)\text{Points}\left(s\right) \end{array}$$ (4)

where *P*^*μ*^(*s*; *x*, *y*) = ∑_*k*_ *x*_*k*_(*x*, *y*)*P*_*k*_(*s*) is the mean probability of possession outcome *s* in location *x*, *y* (c.f. Eq 1), derived from the posterior sample generated by the MCMC algorithm, and Points(*s*) is the true point scoring value of possession outcome *s* (converted try is 6; unconverted try is 4; penalty goal is 2; drop goal is 1; no score is 0). That is, the sum of true points scoring values, weighted by their probability of occurring, was used to estimate the EPV at any given location. When (*x*, *y*) are not specified, we will often omit them in the notation and write EPV to mean the whole map of EPV values on the pitch.

### Modelling procedure

The analysis for this study was conducted at two levels: whole league; and team attacking/defending. First, the whole league model was estimated using the whole league data. For this proof-of-concept study, human-defined priors were used for *P*_*k*_ (Appendix C in S1 Appendix) for the whole league model. These priors were selected after discussion with experts and were informed by previous research [5, 6]. They loosely informed the model that there was a greater chance of points being scored by the end of the possession the closer the location was to the opposition try line.

The 12 team attacking and 12 team defending models used the data subsets described in Section *Data Preprocessing*. The prior distribution parameters *α*_*k*_ were calculated using the possession outcome probabilities from the whole league model posterior distribution. This Bayesian approach allowed the sharing of information between models, which was important in this situation where there was too little data to fit the models independently. Furthermore, it allowed a logical set of priors to be computed (i.e. the whole league possession outcome probabilities, which every team contributed to), from which differences between teams could be evaluated in individual models.

Smooth pitch surfaces were produced for each of the 25 models showing the mean possession outcome probabilities *P*_*k*_ and EPV.

### Analysis of team and player performances

Team performances were evaluated through visual inspection of the possession outcome probability and EPV smooth pitch surfaces. The plots compare the probabilities/EPV of each team attacking/defending model to the whole league model and provide an understanding of areas where a team is more or less likely to generate value at the individual possession outcome or overall EPV level compared to the average team.

Player performances were evaluated using Actual vs Expected (AE) player performance ratings, devised from the EPV values generated by the whole league model. For each action performed by the player, the actual outcome of the possession was compared to the average outcome for the location, i.e., the estimated EPV. These values were summed and divided by the median number of possessions per player’s team per fixture. More precisely, recalling the dataset $D={\left(x_{i},y_{i},s_{i}\right)}_{i=1}^{n}$, we defined

$$\begin{array}{c} \text{Player}\;\text{X}’\text{s}\;\text{AE}\;\text{rating}=\frac{\sum_{i\;\text{such}\;\text{that}\;\text{player}\;\text{X}\;\text{in}\;\text{possession}}\left(s_{i}-EPV\left(x_{i},y_{i}\right)\right)}{\text{Player}\;\text{X}’\text{s}\;\text{team}\;\text{median}\;\text{number}\;\text{of}\;\text{possessions}\;\text{per}\;\text{fixture}}. \end{array}$$ (5)

This choice of the denominator ensured that players from teams who had more possessions within a match were not unduly favoured by the results.

All preprocessing and analysis was completed using bespoke Python scripts (Python 3.7, Python Software Foundation, Delawere, USA) and the PyMC3 v3.11.4 package [15].

## Results

### Whole league model

Of the 99,966 actions included in this study, only 91 occurred in the opposition try area. The EPV_(*x*, *y*)_ of opposition try area actions was much greater than those actions outside of the opposition try area. For example, using centre values, the highest EPV in the field of play was at centre (50, 100), where EPV_(50,100)_ = 1.73, whereas all three opposition try area centres had much greater values (EPV_(*x*,*y*)_ ∈ {3.52, 3.72, 3.16}). Consequently, opposition try area values are removed from all figures as including them would result in colour scales which provide limited insight. The opposition try area actions are included in the player ratings though.

**Fig 2:**
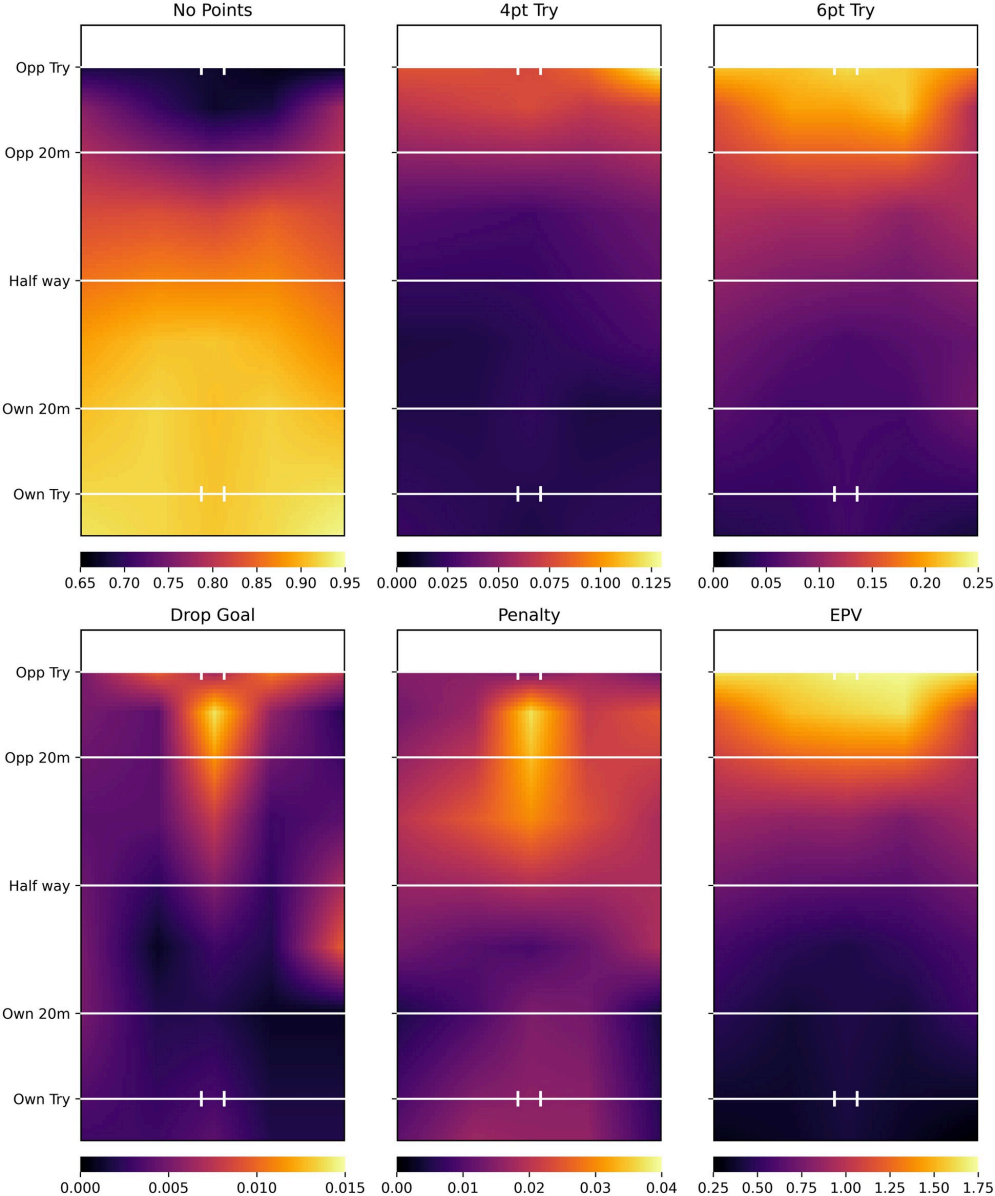
Whole league model mean plot. 4pt Try and 6pt Try refer to unconverted and converted tries respectively. All plots show probabilities, except EPV, which shows points. Smooth pitch surface for possession outcome probabilities is calculated using Eq 1 for each *x*, *y* location on the pitch. The EPV for each location is calculated using Eq 4. Brighter areas represent higher values; colour coding of values differs between graphs.

Fig 2 shows the smooth pitch surface provided by the whole league model mean parameter estimates. There is a greater probability of points being scored by the end of the possession, the closer the location is to the opposition try area. The darker arc on the no points probability surface close to the opposition try line indicates that more points are likely to be scored from central locations than wider locations unless a player is extremely close to the try line. In general, a converted try was more likely to occur at the end of a possession than an unconverted try and a penalty goal was more likely to be scored than a drop goal. The statistical performance of the model is evaluated in Appendix B in S1 Appendix.

### Team attacking and defending models

**Fig 3:**
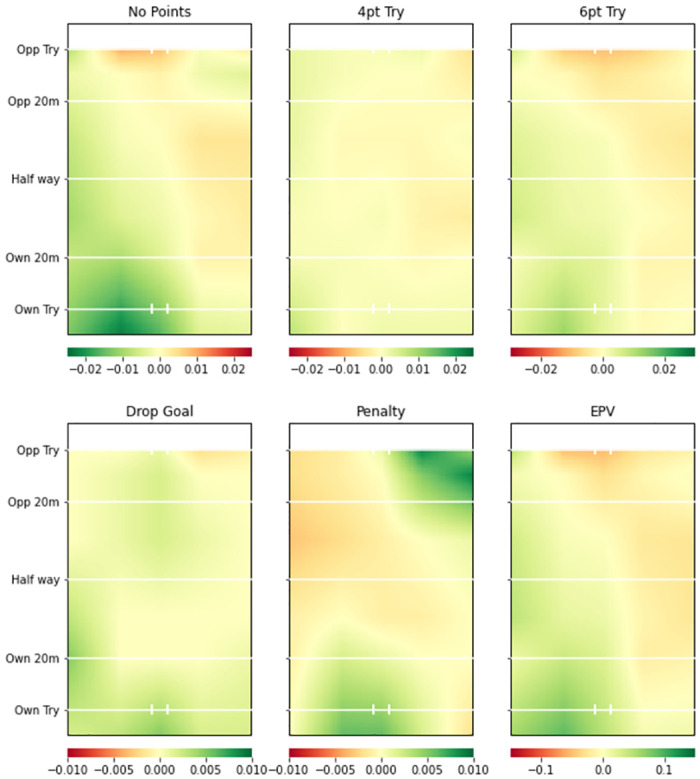
Team A smooth pitch surface plot from team attacking model. Green areas represent higher value for a more favourable possession outcome (i.e. greater probability of all events occurring except No Points) compared to whole league model. 4pt Try and 6pt Try refer to unconverted and converted tries respectively.

Figs 3 (Team A) and 4 (Team B) provide the attacking pitch surface plots from the two teams’ attacking models. There are clear differences between the two plots in different areas across the pitch for all possession outcomes. For example, Team A have a much higher value on the left side of the pitch in all plots except penalty goals. This is mainly shown by a reduced probability of no try, and increased probability of a converted try (6pt) and drop goals on the left side of the pitch. The EPV plot shows the trend even more clearly. Conversely, Team B showed below average attacking prowess. The red areas in all plots except the unconverted try (4pt) indicate that they were more likely to score no points by the end of their possessions and less likely to score any type of points outcome than the average team except unconverted tries.

**Fig 4:**
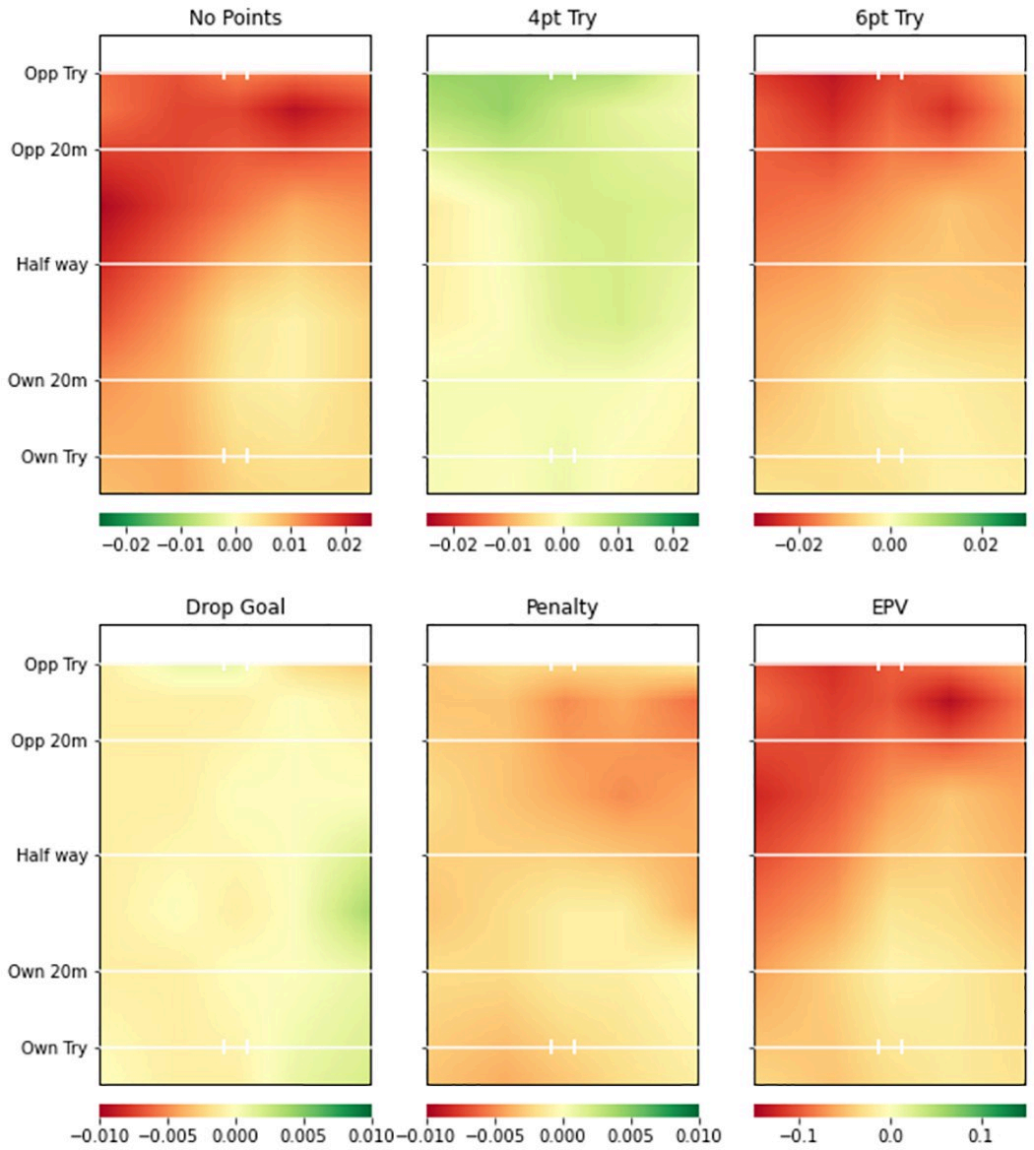
Team B smooth pitch surface plot from team attacking model. Green areas represent higher value for a more favourable possession outcome (i.e. greater probability of all events occurring except No Points) compared to whole league model. 4pt Try and 6pt Try refer to unconverted and converted tries respectively.

**Fig 5:**
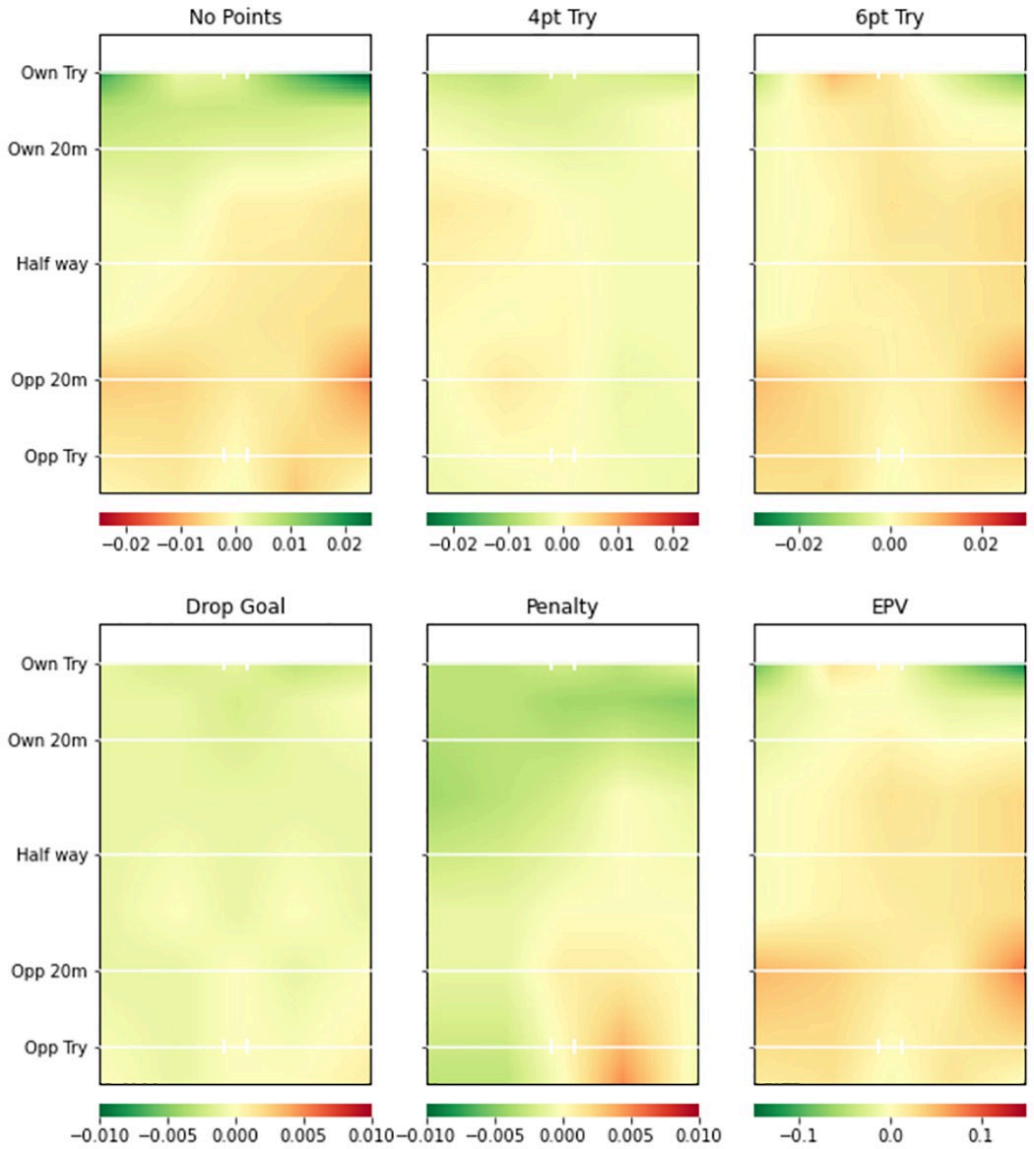
Team A smooth pitch surface plot from team defending model. Green areas represent higher value for more favourable events (i.e. greater probability of all events occurring except No Points) compared to whole league model. 4pt Try and 6pt Try refer to unconverted and converted tries respectively.

Figs 5 (Team A) and 6 (Team B) provide the defending plots from the same two teams’ defending models. Clear differences between the two teams are again visible. Team A has a greater probability than the average team of conceding points up until their own 20m line (shown by the reddish areas on the EPV plot and the no points plot), but has a reduced probability of conceding any points outcome on their own try line in the left and right corners of the pitch. Team B is considerably better than average at defending in all areas except the probability of the opposition team scoring penalty goals.

**Fig 6:**
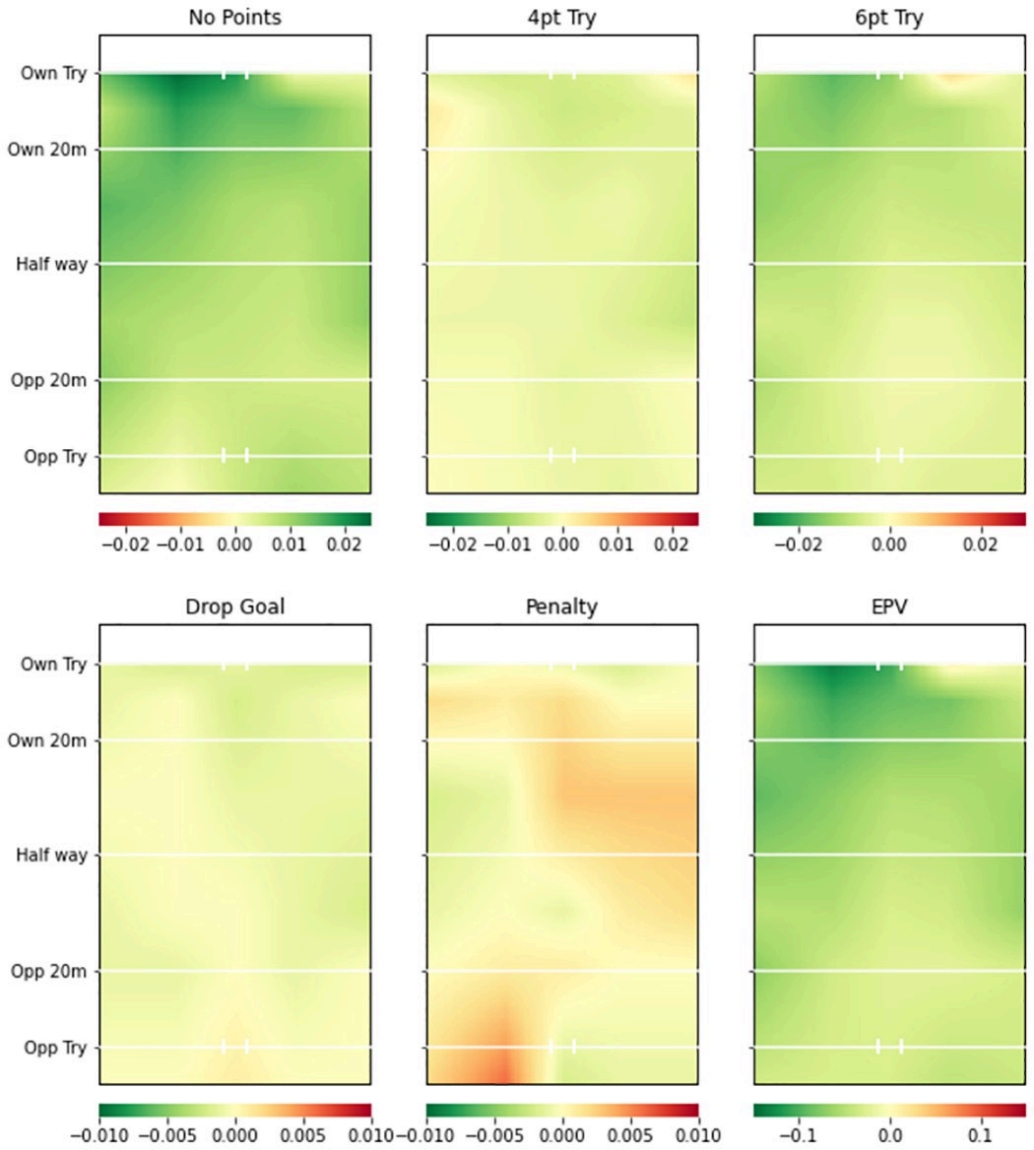
Team B smooth pitch surface plot from team defending model. Green areas represent higher value for more favourable events (i.e. greater probability of all events occurring except No Points) compared to the whole league model. 4pt Try and 6pt Try refer to unconverted and converted tries respectively.

### Player ratings

Table 2 provides player ratings for the 20 best players in the 2021 Super League season according to the AE ratings. A selection of traditional summary statistics (tries, try assists, metres and goals kicked) are provided so the player AE ratings can be compared to these readily available statistics. A wide range of positions was present within the top 20 players, as well as a wide range of scoring profiles based on the four traditional summary statistics provided.

**Table 2:** Top 20 player ratings as assessed by the AE ratings (Eq 5). Tries, Try Assists, Metres and Goals are provided as references of statistics currently provided for player performances. To protect anonymity, reference statistics are provided as T-5 (count of statistic within the top 5 players); T-10 (within the top 10 players), T-20 (within the top 20 players) and 20+ (outside the top 20 players).

Player	Position	AE Rating	Tries	Try Assists	Metres	Goals
276	Full Back	8.21	T-20	T-5	20+	20+
19	Winger	6.67	T-5	20+	T-5	20+
6335	Stand-off	6.35	20+	20+	20+	T-5
1004	Scrum Half	6.10	20+	20+	20+	20+
433	Full Back	4.96	20+	T-10	20+	T-5
158	Winger	4.82	T-5	20+	T-10	20+
188	Centre	4.78	T-5	20+	T-5	20+
1249	Hooker	4.37	20+	20+	20+	20+
92	Scrum Half	3.91	20+	T-5	20+	20+
1281	Loose Forward	3.86	20+	20+	20+	20+
406	Loose Forward	3.82	20+	20+	20+	20+
371	Winger	3.37	T-20	20+	T-20	20+
8	Prop	3.33	20+	20+	T-10	20+
282	Second Row	3.28	20+	20+	20+	20+
20528	Winger	3.11	T-5	20+	T-5	20+
22852	Full Back	3.10	T-10	T-20	20+	20+
5	Winger	3.08	T-20	20+	20+	20+
26	Hooker	2.96	20+	20+	20+	20+
440	Loose Forward	2.88	20+	20+	20+	20+
988	Stand-off	2.81	20+	20+	20+	20+

## Discussion

The primary aim of this study was to introduce a novel Bayesian Mixture Model approach to the development of an EPV model in rugby league, which could produce a smooth pitch surface and estimate individual possession outcome probabilities. A secondary aim of the study was to show how the model could be used to identify differences in teams’ attacking and defending performances and evaluate player performances. Both these aims were achieved.

### The Bayesian Mixture Model approach

The key contribution of this study is the introduction of a Bayesian Mixture Model approach to EPV model development. This approach produced a smooth pitch surface of individual possession outcome probabilities in rugby league despite the sport’s characteristics of low data availability and multiple possession outcomes (100,000 data points and 5 possession outcomes versus 14.4 million data points and 2 possession outcomes for [4] in football). The Bayesian Mixture Model worked by assigning a set of centres across the pitch and using the proximity of a location to these centres to continuously model changes in scoring probabilities across the pitch, improving upon the zonal approaches previously employed [1, 5, 6]. The Bayesian approach allowed prior distributions to provide the model with an understanding of possible parameter values before the fitting process began and allowed information sharing between the different levels of analysis (i.e. using the whole league model posterior distribution to calculate the team attacking/defending models’ prior distributions).

Fig 2 plots the smooth pitch surfaces provided by the whole league model. At the EPV level, the results are similar to previous studies, which suggest that there is greater value in areas closer to the opposition try line [5, 6] and more centrally [6]. However, at the individual possession outcome level, much greater insights are obtained. For example, the importance of central areas to drop goal and penalty goal success is clearly shown. Similarly, there is an increase in the probability of unconverted tries being scored on the far right of the pitch. Neither of these insights would be possible without the estimation of individual possession outcome probabilities.

The Bayesian Mixture Model approach provides an exceptional amount of flexibility, both with respect to the EPV measure and the probabilities it could estimate. In this study, the EPV was defined as the weighted average of all possession outcomes, but future studies may wish to consider whether using try/no try probabilities can provide additional insights. Similarly, the Mixture Model approach can handle more probabilities if required. For example, by preprocessing the data in a different way and providing categories for the next scoring outcome (converted try, unconverted try, penalty goal and drop goal for both teams, alongside no score), it would be possible to estimate 9 scoring outcome probabilities and use these to develop a “next scoring outcome” EPV measure.

### Team level insights

Figs 3 and 4 provide attacking pitch surfaces for two separate teams. It is clear from visual inspection of the plots that strategic insights regarding both teams’ performances can be generated. For example, Team B was particularly poor at attacking across the pitch. Conversely, Team A was more likely to score tries on the left side of the pitch, but more likely to score a penalty goal on the right side of the pitch. Figs 5 and 6 provide defensive pitch surfaces for the same two teams. Again, the plots provide valuable insights. It can be deduced from Fig 5 that Team A are much more likely to concede points from possessions beginning outside their 20m. However, if actions occur close to their try line, they are able to defend them particularly well in the corners of the pitch. Team B was excellent defensively across the pitch. Indeed, the penalty goal plot suggests that opposition teams were more likely to try and score penalties against them than the average team, potentially because they were unable to break down the defence and score tries. The ability of the model to identify these differences between teams is extremely valuable with respect to developing tactical strategies for upcoming matches (e.g., understanding where to attack or defend against a specific team). It would now be beneficial to develop a methodology through which differences in these attacking and defensive probabilities or values can be objectively identified.

### AE player ratings

Table 2 provides the top 20 players across the 2021 Super League season based on the AE player ratings. The rating denotes the points contribution actions taken by a player provided per match (e.g. player 276’s actions contributed 8.21 points per match to their team’s overall points count). A wide variety of positions and traditional statistic profiles are included. Some of these players scored more tries, others were better at providing try assists or kicking goals; others did not excel in any of the summary statistics provided. This ability to understand valuable players from different positions and across different summary statistic profiles differs from previous research [12], which predominantly valued players who attempted try scoring actions. However, players who were involved in a large number of actions in possessions which resulted in no try being scored due to the location they began in (e.g. most of the scrum halves) were valued poorly. It may therefore be appropriate to consider models which evaluate a different set of outcomes, or a different calculation of the player ratings, to value these positions accurately. Regardless, the results of this study provide a framework through which players can be valued objectively and transfer targets can be identified for clubs attempting to improve their performance or replace outgoing players.

### Limitations and future directions

The model described in this paper significantly advances the approaches used in previous studies in rugby league [5, 6]. It provides a flexible methodology, which could be used to generate EPV models in any sport where data is not readily available but is subject to two key limitations. The first of these is that it only considers event-level data, so there is limited context surrounding the value of the locations. Therefore, if a player is stood with 5 defenders directly in front of him or no defenders directly in front of him, his location would be valued the same. Secondly, the model does not yet incorporate the auto-correlation present within possession sequences. Although the impact of this on parameter estimates is likely to be limited due to the length of the possession sequences, it is still a limitation of the model. Alongside the future directions indicated in the sections above, future studies may wish to address these limitations when appropriate data is available.

## Conclusion

In this paper, a novel Bayesian Mixture Model approach to the estimation of an EPV Model was proposed in rugby league. The model was able to provide a smooth pitch surface and estimate the probability of individual possession outcomes occurring. Insights into player and team performances were derived from the model showcasing its ability to provide valuable information for upcoming fixtures and player recruitment. This information can be used to develop strategies to win upcoming matches and to produce lists of transfer targets to improve the teams’ performances. Given appropriate data preprocessing and modification of possession outcome categories, the model could be adapted to any invasion sport’s requirements.

## Supporting information

S1 Appendix Further details on data processing, execution of research and analysis of results. (ZIP)
