# Supplementary material for: A Bayesian Mixture Model approach to expected possession values in rugby league
Source: PLoS One. 2024 Nov 21;19(11):e0308222. doi: 10.1371/journal.pone.0308222 (PMC11581272; doi:10.1371/journal.pone.0308222)
Supplement: S1 Appendix — (ZIP) [file pone.0308222.s001.zip › S1 Appendices.pdf]

S1 Appendices for
A Bayesian Mixture Model approach to expected possession values in rugby league

Thomas Sawczuk, Anna Palczewska, Ben Jones, Jan Palczewski

A Data Preprocessing

This section provides further details surrounding the preprocessing steps taken to reduce the dataset from 557,050 observations to the 99,966 action locations used in the analysis.

To facilitate the data filtering process, actions were split into 23 preprocessing categories (Table 1). Only actions taken by the attacking team were required for analysis so only actions belonging to the "move team"; "move self"; "catch kick"; "kick position"; "move team error"; "move self error"; "loose ball" and "kick goal" categories were used. Using only this subset of actions was necessary as a player completing a "move self" action could also be given a "run action" descriptor if they ran past an opponent, thus providing two entries for the same action (i.e. a multiple action coding). Additionally, incomplete/unsuccessful actions from any of these categories were only included if they resulted in the end of a possession. For example, an unsuccessful attempt at a loose ball collection or an unsuccessful attempt to intercept a pass by the defensive team were not included as these events were not successfully completed and including them would have unduly affected the chain of possession. However, an incomplete pass, or a dropped catch from a pass by the attacking team were included as these actions resulted in a change of possession.

As the study used the location of actions, it was important that the same location was not coded on more than one occasion for different actions completed consecutively within the same sequence. For example, if a player caught the ball and decided to run in the same location, Opta would code each action as an individual observation. Including both locations in the dataset could bias the results so multiple location codings were merged into a single location by removing the second consecutive location from the dataset indiscriminately. This indiscriminate removal could only be performed because the type of action (e.g. run, pass, kick) was not considered in this analysis.

Table 1. Preprocessing categories and actions included within them in this study

Category	Events
Auxiliary Information	Front Marker, Back Marker, Video Ref, Interchange, HIA, Stoppage
Generic Descriptor	Other Error, Try cause, Line Break Involvement, Line Break Assist, Break Cause, Tackle Break, Opp Error, Passing Move, Close Range, Error, Try Involvement, Long Range, Individual Effort, Other, Sin Bin Out, Yellow, Sin Bin Return To Field, Grounding, Contest, Sent Off Out, Red, Touchline/Deadball, Onside, On Report
Restart Actions	50m Restart, Goal Line Drop Out, 20m Restart
Move Self	Restart Run, Evasion, Hitup, Kick Return, Line Engaged, Ruck Run, Dummy Half, Run, Line Not Engaged
Move Team	Complete, Short - Crossfield, Short - Grubber, Own Player, Break, Short - Banana, To Ground, Short - Bomb, Short - Chip, Try
Kick Goal	Conversion, Penalty Goal, Field Goal
Kick Position	Long - To Opposition, Good, Long - To Open, Long - 40-20, Long - Touch
Catch Pass	Simple Receipt, Jump Catch
Catch Kick	Kick Receipt, Restart Receipt
Loose Ball	Defensive Cleanup, Attacking Cleanup, Attempted Intercept, Contestable Cleanup, Interception
Tackle	Made, Dominant, Offload To Ground, Turnover Ball Split, Forced Within In Goal, Stolen, Turnover Into Touch, Offload
Missed Tackle	Bumped Off, Stepped, Positional, Outpaced, Try Conceded
Run Action	Dummy Pass, Half Break, Line Break, Carried Dead Ball, Forced Into In Goal, Carried In Touch
Play-The-Ball	Lost, Won, Interrupted
Attacking Descriptor	Kick Line Break, Try Assist, From Kick, From Penalty, From Line
Defensive Descriptor	Kick Pressure
Move Self Error	Dropped Ball Unforced, Ball Jolted, Lost Ball Forced
Move Team Error	Not Out, Failure To Find Touch, Incomplete, Off Target, Forward Pass, Forward, Kick Error, Bad Offload, To Opposition, Bad Pass, PTB Fumble, Intercepted
Catch Error	Accidental Knock On, Falcon
Penalty Conceded	Defence, Penalty, Inside 10m, Attack, Ruck Infringement, Foul Play, Double Movement, Obstruction
Defensive Play	Flop, Kick Not Defused, Kick Defused, Charge Down, Attempted Steal
Off The Ball	Decoy, Support Run, Kick Shield, Kick Shepherd, Kick Chase

B Statistical performance of the estimated model

Bayesian analysis allows the statistical performance of models to be evaluated through analysis of the posterior distribution. In the main text, the mean of the posterior distribution of probabilities was presented and analysed. Here the variability of the posterior distribution is analysed by considering the standard deviation of the posterior distribution of the probability of each possession outcome at each location.

The standard deviation of the posterior distribution of possession outcome probabilities P_k^σ is used to measure the uncertainty within the parameter estimates for any x, y location. For an outcome s at location x, y, the standard deviation of the posterior distribution is approximated by

$$P^\sigma(s; x, y) = \sqrt{\sum_k z_k(x, y) P_k^\sigma(s)^2},$$

where $P_k^\sigma(s)$ is the standard deviation of posterior probability of s at centre k estimated by the sample standard deviation of the posterior sample generated by the MCMC algorithm.

The standard deviation of the posterior EPV estimate is approximated by

$$\text{EPV}^\sigma_{(x,y)} = \sqrt{\sum_{s \in S} P^\sigma(s; x, y)^2 \text{Points}(s)^2}$$

Figure 1 plots the variability in the smooth pitch surface for each possession outcome using the whole league model. In all possession outcome plots, there is greater variability in the wider areas of the pitch, which is accompanied by a lower density of actions in those areas in the KDE plot. This variability is particularly large for the penalty goal probabilities in wide areas. Similarly, there is increased variability in try/no try probabilities in both corners of the pitch on the opposition try line. In all circumstances, the greater uncertainty is unsurprising given that a greater density of actions took place within the centre of the pitch, but practitioners should interpret the findings in these areas with greater caution relative to the central areas of the pitch where more data is available.

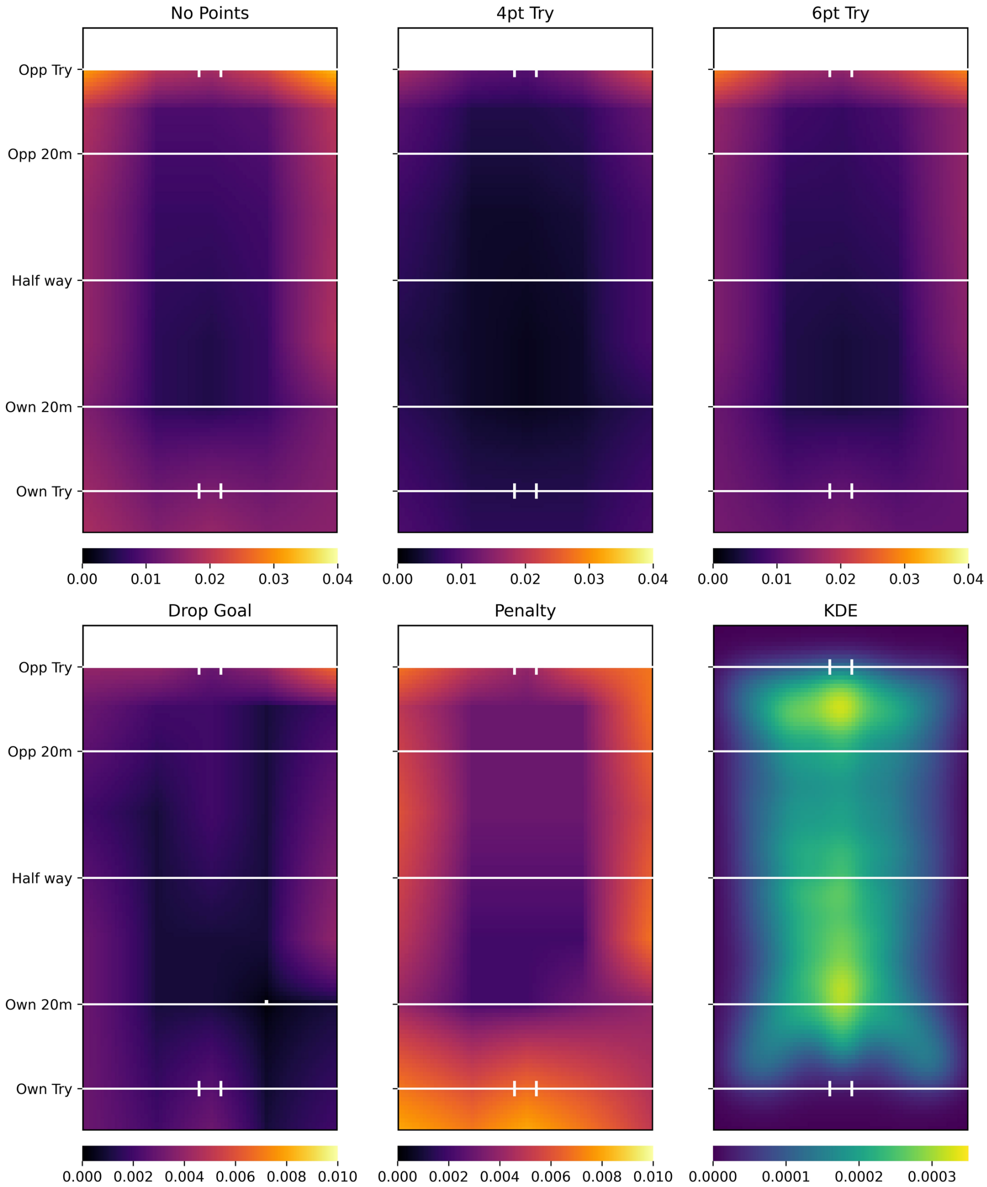

Fig 1. Whole league model EPV standard deviation plot. 4pt Try and 6pt Try refer to unconverted and converted tries respectively. Kernel Density Estimation plot of dataset is provided to give an understanding of location densities. Brighter colours indicate higher values.

C Whole league model priors

Table 2 displays the prior distribution parameters α_k for each centre k used in the estimation of the whole league model. These priors loosely informed the model that there was a greater chance of points being scored by the end of the possession the closer the location was to the opposition try line. They could have been refined further by indicating that points were more likely to be scored in central areas as suggested by [1], but a more loosely informative prior was selected as it was unclear if the trend would be shown for each individual possession outcome.

D Team model priors

Table 3 displays prior distribution parameters α_k for each centre k. These values were calculated by fitting a Dirichlet distribution to a sample from the posterior distribution of the whole league model using maximum likelihood estimation. All team attacking and defending models used this prior distribution.

References

1. Sawczuk T, Palczewska A, Jones B. Development of an expected possession value model to analyse team attacking performances in rugby league. PLOS ONE. 2021;16(11):1–14.

Table 2. Prior α values for whole league Bayesian model. Centre coordinates are provided, alongside α values for each possession outcome. 'C.Try' refers to converted try (6 points) and 'U.Try' refers to unconverted try (4 points).

Centre	No Try	Drop Goal	Penalty	U.Try	C.Try
(0, -10)	90	1	1	4	4
(20, -10)	90	1	1	4	4
(35, -10)	90	1	1	4	4
(50, -10)	90	1	1	4	4
(70, -10)	90	1	1	4	4
(0, 20)	90	1	1	4	4
(20, 20)	90	1	1	4	4
(35, 20)	90	1	1	4	4
(50, 20)	90	1	1	4	4
(70, 20)	90	1	1	4	4
(0, 35)	85	1	3	5	6
(20, 35)	85	1	3	5	6
(35, 35)	85	1	3	5	6
(50, 35)	85	1	3	5	6
(70, 35)	85	1	3	5	6
(0, 65)	80	1	3	7	9
(20, 65)	80	1	3	7	9
(35, 65)	80	1	3	7	9
(50, 65)	80	1	3	7	9
(70, 65)	80	1	3	7	9
(0, 90)	75	1	3	9	12
(20, 90)	75	1	3	9	12
(35, 90)	75	1	3	9	12
(50, 90)	75	1	3	9	12
(70, 90)	75	1	3	9	12
(0, 100)	70	1	3	10	15
(20, 100)	70	1	3	10	15
(35, 100)	70	1	3	10	15
(50, 100)	70	1	3	10	15
(70, 100)	70	1	3	10	15
(0, TRY)	35	1	1	28	35
(35, TRY)	35	1	1	28	35
(70, TRY)	35	1	1	28	35

Table 3. Prior distribution parameters α_k for team attacking and defending models. Centre coordinates are provided, alongside α values for each possession outcome. 'C.Try' refers to converted try (6 points) and 'U.Try' refers to unconverted try (4 points). Values rounded to 2 decimal places for brevity.

Centre	No Try	Drop Goal	Penalty	U.Try	C.Try
(0, -10)	184.72	0.72	2.01	4.97	6.43
(20, -10)	345.31	1.07	6.38	7.45	14.88
(35, -10)	280.81	1.37	4.63	5.08	16.00
(50, -10)	365.08	0.95	6.30	7.90	15.67
(70, -10)	230.12	0.77	1.28	5.40	6.11
(0, 20)	371.56	2.28	1.87	7.08	27.40
(20, 20)	1559.21	2.73	14.96	28.85	86.65
(35, 20)	2815.84	6.64	44.81	67.00	168.66
(50, 20)	1599.20	1.03	22.07	25.46	94.59
(70, 20)	393.10	0.73	1.75	6.92	35.35
(0, 35)	373.60	2.10	5.30	6.51	33.39
(20, 35)	1707.17	2.05	18.37	30.07	121.44
(35, 35)	2636.37	8.50	24.50	59.31	162.86
(50, 35)	1673.02	3.58	21.79	44.56	116.69
(70, 35)	310.63	3.68	6.61	10.50	25.85
(0, 65)	439.66	2.03	10.38	17.21	63.22
(20, 65)	2189.67	10.97	65.76	75.37	294.36
(35, 65)	2546.81	26.14	94.46	84.30	355.25
(50, 65)	1726.64	6.24	49.69	68.55	197.80
(70, 65)	360.60	1.82	8.02	18.25	51.31
(0, 90)	413.37	2.70	6.90	35.62	83.31
(20, 90)	1356.56	8.21	32.70	139.25	384.54
(35, 90)	2150.80	43.80	116.69	241.11	636.41
(50, 90)	1437.66	12.84	43.18	142.16	463.60
(70, 90)	365.55	0.91	11.60	34.77	50.19
(0, 100)	154.63	1.15	3.38	18.26	50.01
(20, 100)	370.25	5.46	8.15	42.98	116.60
(35, 100)	427.75	3.94	7.62	47.37	146.33
(50, 100)	299.46	4.83	7.87	40.45	99.93
(70, 100)	121.38	1.30	2.36	24.68	34.07
(0, TRY)	36.62	0.98	0.95	33.38	46.66
(35, TRY)	43.45	0.97	0.99	29.80	68.50
(70, TRY)	42.17	1.00	1.02	30.12	39.13
